# A simple, rapid method to isolate salt glands for three-dimensional visualization, fluorescence imaging and cytological studies

**DOI:** 10.1186/1746-4811-6-24

**Published:** 2010-10-19

**Authors:** Wee-Kee Tan, Tit-Meng Lim, Chiang-Shiong Loh

**Affiliations:** 1 Department of Biological Sciences, National University of Singapore, 14 Science Drive 4, 117543, Singapore; 2 NUS Environmental Research Institute, National University of Singapore, 5A Engineering Drive 1, T-Lab, #02-01, 117411, Singapore

## Abstract

**Background:**

Some plants inhabiting saline environment remove salts via the salt glands embedded in the epidermal tissues. Cytological studies of salt glands will provide valuable information to our understanding of the secretory process. Previous studies on salt gland histology relied mainly on two-dimensional microscopic observations of microtome sections. Optical sectioning properties of confocal laser scanning microscope offer alternative approach for obtaining three-dimensional structural information of salt glands. Difficulty in light penetration through intact leaves and interference from neighbouring leaf cells, however, impede the acquiring of good optical salt gland sections and limit its applications in salt gland imaging. Freeing the glands from adjacent leaf tissues will allow better manipulations for three-dimensional imaging through confocal laser scanning microscopy.

**Results:**

Here, we present a simple and fast method for the isolation of individual salt glands released from the interference of neighbouring cells. About 100-200 salt glands could be isolated from just one cm^2 ^of *Avicennia officinalis *leaf within hours and microscopic visualization of isolated salt glands was made possible within a day. Using these isolated glands, confocal laser scanning microscopic techniques could be applied and better resolution salt gland images could be achieved. By making use of their intrinsic fluorescent properties, optical sections of the gland cells could be acquired without the use of fluorescent probes and the corresponding three-dimensional images constructed. Useful cytological information of the salt gland cells could also be obtained through the applications of fluorescent dyes (e.g., LysoTracker^® ^Red, FM^®^4-64, Texas Red^®^).

**Conclusions:**

The study of salt glands directly at the glandular level are made possible with the successful isolation of these specialized structures. Preparation of materials for subsequent microscopic observations of salt glands could be achieved within a day. Potential applications of confocal fluorescence microscopic techniques could also be performed using these isolated glands. Experiments designed and targeted directly at the salt glands were explored and cytological information obtained herein could be further incorporated towards the understanding of the mechanism underlying secretion in plant salt glands.

## Background

Salt glands are specialized adaptive structures found predominantly on the leaves and stems of halophytic species [1,2]. They are considered to be efficient desalination devices capable of removing salts from the plant tissues via an energy-dependent secretion process [1]. Earlier research on the histological and ultrastructural studies of salt glands had relied mainly on microtome sections of leaf tissues [1,3-9]. Preparation of materials for conventional microtome sectioning, however, is laborious and time consuming as it involves fixation of plant tissues in appropriate fixatives followed by dehydration in alcohol series and infiltration with wax prior to embedding the samples in paraffin wax [10,11]. Microtome sectioning of paraffin-embedded leaf samples to obtain the desired plane of sections containing the salt glands is also challenging, especially if the salt glands are sparsely distributed. In addition, observations of microtome sections are usually confined to two-dimensional imaging involving brightfield microscopy or electron microscopy as far as ultrathin microtome sections are concerned. Technical difficulty in obtaining a complete series of microtome sections [11] limits the use of microtomy techniques in gathering three-dimensional structural information of the salt glands. We therefore look into alternative ways for the observations of the structural organization of salt glands in greater detail and at higher resolution.

Optical sectioning microscopy, such as confocal laser scanning microscopy, is a powerful tool for biological investigation, and is currently one of the most adequate and straightforward three-dimensional imaging methods for obtaining high resolution images up to single-cell level in plant biology [12,13]. In contrast to conventional wide-field fluorescent and light microscopy, images taken using confocal laser scanning microscopes showed much improved clarity with the successful elimination of out-of-focus background noise via the pinhole [11,12,14]. A series of optical sections of intact specimens can thus be collected through the use of confocal laser scanning microscopy and the corresponding three-dimensional views reconstructed using computer-aided softwares [11,12].

Optical sectioning of intact specimens, however, has its limitation as it is difficult to obtain deep sections (>50-100 μm) from intact tissues, especially for uncleared botanical specimens such as the leaves that tend to be more opaque in nature [11,14]. Even though the salt glands of *Avicennia officinalis *in the current study are distributed on the epidermal layers of the leaves, they are either sunken in deep pits or depressions for those found on the adaxial epidermal surfaces, or are intermingled with trichomes on the abaxial surfaces [4,15]. Penetration of light through intact leaves to obtain the whole z series of the salt glands might thus be difficult and the presence of neighbouring epidermal cells or trichomes on the leaf surfaces further interfere with acquiring good optical salt gland sections. In addition, the presence of chloroplast-rich mesophyll and palisade cells in the leaves limits the use of secondary fluorochromes since red autofluorescence of the chlorophyll in these cells will obscure the signals obtained from the salt glands on the epidermal layers. Alternative approach to acquiring the entire z-stacks of the salt glands directly from intact leaves will need to be explored. Obtaining specimens that can free the salt glands from the interference of neighbouring cells will be ideal for further manipulations and imaging through confocal laser scanning microscopy.

In this paper, a method to isolate large numbers of individual salt glands as complete entities devoid of neighbouring leaf tissues is presented. Observations of isolated salt glands through brightfield, fluorescent as well as confocal laser scanning microscopy are possible, with each revealing the structural organizations of the salt glands to different degree of clarity. In contrast to visualization of salt glands directly from intact leaves, optical sectioning of the isolated salt glands showing both the top and side views is possible without the need to prepare paradermal and transverse leaf sections separately using classical microtomy techniques. The intrinsic autofluorescent nature of the salt glands also allows optical sections to be taken and subsequent three-dimensional images of the salt glands to be constructed without fluorescent dye applications.

## Results and Discussion

### A simple method for salt gland isolation

**Figure 1:**
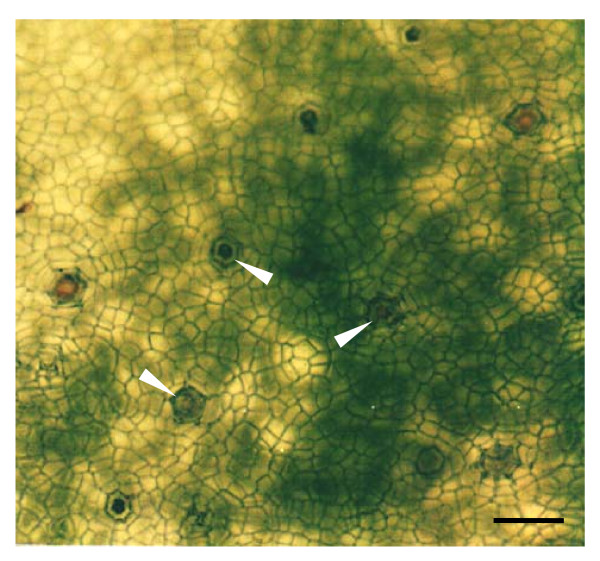
**Paradermal view of the salt glands of *A*. *officinalis***. Adaxial epidermal peel was obtained through an enzymatic approach. The top view of salt glands (arrows) can be viewed directly from the isolated epidermal peel using brightfield microscopy. Note the presence of chlorophyll-containing cells (dark green patches) underneath the epidermal peel that tend to obscure the salt gland images. Bar = 100 μm.

Recent imaging trend prefers less invasive methods aiming at keeping the cells and tissues intact and alive [11,14]. The degree of light penetration through whole-mount specimens, however, limits the application of state-of-the-art microscopic techniques, such as confocal laser scanning microscopy, in obtaining deep optical sections with good cellular details from living plant tissues [11,12,14]. To solve the problem of direct visualization of salt glands from intact leaves without any clearing treatments, we enzymatically isolated adaxial epidermal peels where the salt glands are found for subsequent microscopic studies (Figure 1). Through this approach, the thickness of the leaf sections containing the salt glands was reduced, rendering paradermal microscopic observations of the salt glands possible without the need to prepare microtome leaf sections. Red autofluorescence of the chlorophyll in the mesophyll and palisade cells underneath the salt glands, however, obscured the structural observations of the salt glands on the epidermal layers when the samples were viewed under fluorescent or confocal laser scanning microscopes. Alternative approach will be required to obtain good optical sections of the salt glands using confocal laser scanning microscopy.

**Figure 2:**
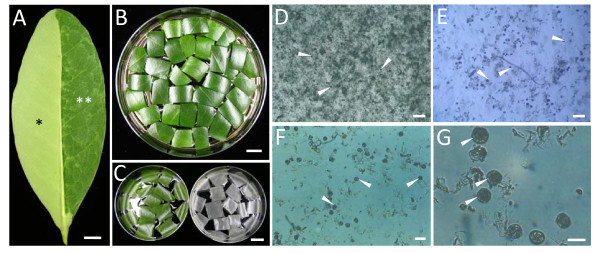
**A simple method for the isolation of salt glands from *A*. *officinalis***. (A) The lower epidermal layer (*) of the leaf was removed with a razor blade to reveal the mesophyll-palisade cell layers (**) prior to enzyme digestion. Bar = 1 cm. (B, C) The leaf with the lower epidermal layer removed were cut into strips and incubated in enzyme solution (B) to obtain the adaxial epidermal peels (C). The epidermal peels appear green (C; left image) due to the presence of mesophyll-palisade cells underneath the epidermal layer, which can be easily removed to obtain peels devoid of chlorophyll-containing cells (C; right image). Bars = 1 cm. (D-G) Salt glands (arrows) were then released through the grinding of these peels (C; right image) followed by a series of purification processes, which include filtering through a 100 μm (pore size) cell strainer (D). The isolated salt glands were finally collected on a 20 μm (pore size) cup filcon (E). All isolated salt glands (D-G) were observed under Olympus inverted light microscope (IMT-2), with close-up observations of purified salt glands viewed under different magnifications (F, G). Bars in D and E = 200 μm, bar in (F) = 80 μm, and bar in (G) = 40 μm.

This report presents a simple and fast isolation method that enables us to obtain large numbers of isolated salt glands that are freed from the interference of neighbouring epidermal cells and the mesophyll-palisade cell layers. (Figure 2; see Methods for full descriptions). By removing the abaxial epidermal cell layers from the leaf (Figure 2A) and incubating the resulting leaf tissues in an enzyme solution (Figure 2B), the mesophyll-palisade tissues can be easily detached from the adaxial epidermal peels after an hour of enzymatic digestion. Remnants of mesophyll-palisade cells (as observed by their green appearance due to the presence of chloroplasts) underneath the adaxial epidermal peels can be removed with ease (Figure 2C) to minimize contamination with other cell types prior to salt gland isolation. The salt glands can then be released through the grinding of these epidermal peels.

**Figure 3:**
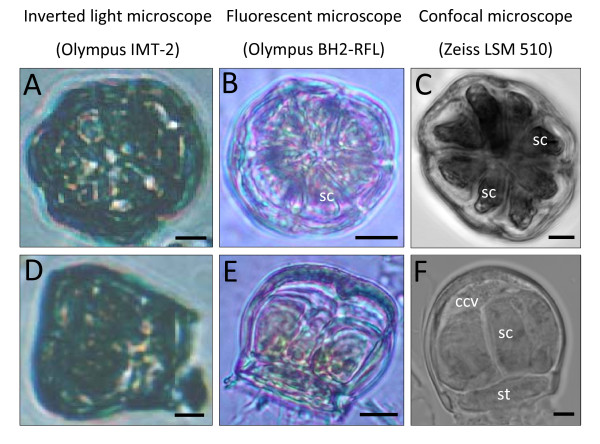
**Microscopic studies of isolated salt glands of *A*. *officinalis***. Top view (A-C) and side view (D-F) of isolated salt glands. Note the increase in the degree of resolution of images presented from left to right, with confocal microscopic images showing the clearest morphological details of the isolated salt glands. sc: secretory cell; st: stalk cell; ccv: subcuticular collecting cavity. Bars = 5 μm.

Using this method, the salt glands of *A*. *officinalis *can be successfully isolated within 3-4 hours (Figure 2D-G) and observations of salt glands could be achieved within the same day. Approximately 2 × 10^4 ^- 3.5 × 10^4 ^of salt glands could be obtained for each isolation, i.e., about 100-200 salt glands were harvested from one cm^2 ^of leaf tissues used during the isolation process. Each isolated salt gland was found to be approximately 30-40 μm in diameter (Figure 2G and Figure 3), which is comparable to the salt glands of other *Avicennia *species reported so far [1,2,5,15].

Microscopic views of individual salt glands revealed a general salt gland structure (Figure 3), with confocal microscopic images (Figure 3C, F) showing unambiguously the cell number within each salt gland. Both the top and side views of the salt glands could be observed from each session of salt gland isolation, in contrast to conventional microtome sectioning where paraffin-embedded samples of different orientations need to be prepared separately to obtain paradermal and transverse leaf sections. A total of 8 radially arranged secretory cells was observed from the top view of the isolated salt gland (Figure 3C). From the side view (Figure 3F), 3 secretory cells were observed and a single stalk cell was positioned at almost perpendicular to the group of secretory cells above it. The secretory cells and stalk cell were enclosed by a cuticle layer, which formed the general outline of the salt gland. A subcuticular collecting cavity above the outermost secretory cells and below the top cuticle layer was also detected (Figure 3F).

The salt glands of *A*. *officinalis *thus belong to the Type III salt gland as categorized by Thomson et al. [1], which are multicellular comprising a characteristic disc-shaped stalk or basal cell subtended by one or more sub-basal (collecting) cells below and several secretory cells above. The basic descriptions of the salt glands reported herein were similar to the ultrastructures of the salt glands of other *Avicennia *and *Acanthus *species studied [4,5,7,8,15-17].

### Autofluorescence of salt glands

**Figure 4:**
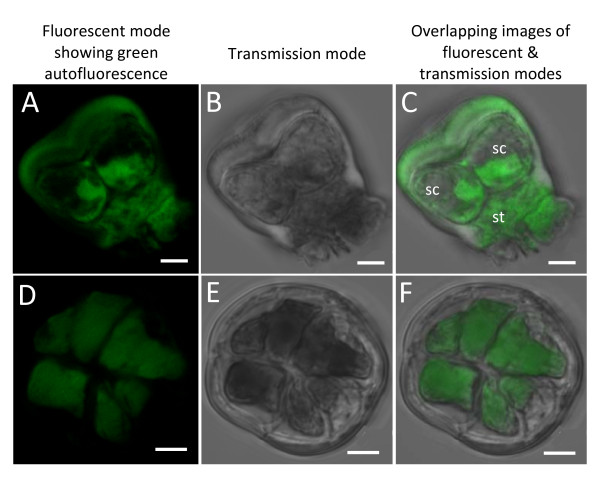
**Isolated salt glands of *A*. *officinalis *showing their intrinsic autofluorescent nature**. (A-C) Side view of salt gland. Note the presence of 2 secretory cells (sc) above the stalk cell (st). (D-F) Top view of salt gland with a total of 7 secretory cells (green patches) observed. The outlines of these cells were emphasized by their autofluorescent nature. Excitation wavelength was 488 nm (8-10%) and optical sections were taken using Zeiss LSM 510. Images from optical sectioning were stacked to give a clearer picture of the salt glands. Bars = 5 μm.

**Figure 5:**
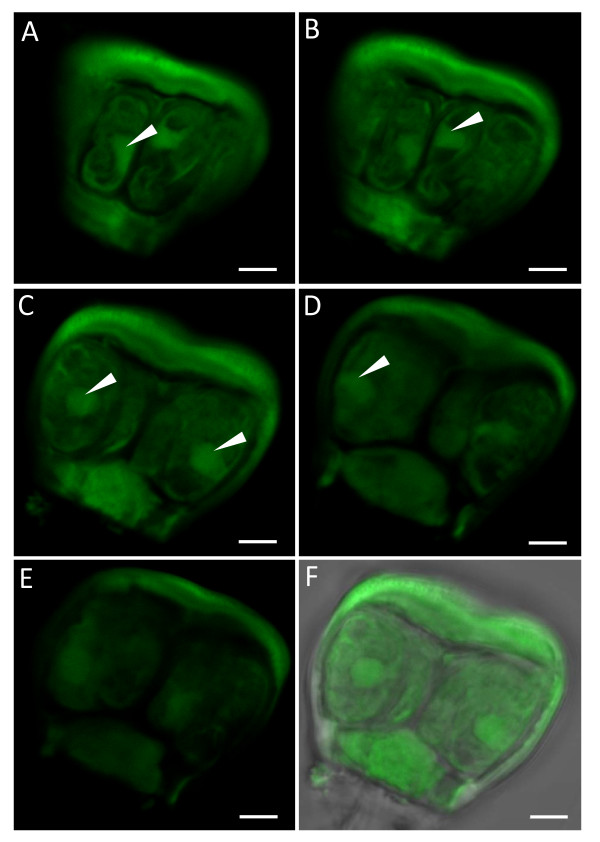
**Optical sections of an isolated salt gland of *A*. *officinalis***. Sections (A-E) were taken in the fluorescent mode to show the side views of a salt gland. Details within cells could be observed due to the green autofluorescent nature of the salt gland. Note the presence of strongly autofluorescent structures (arrows) in the middle or periphery of some secretory cells. (F) Corresponding bright-field image of (C) taken together with overlapping image recorded simultaneously in the transmission and fluorescent modes. Optical sections were taken with Zeiss LSM 510. Excitation and emission wavelengths were 488 nm (10%) and >530 nm, respectively. Bars = 5 μm.

Green autofluorescence was observed from the isolated salt glands when excited by blue light (wavelength of 488 nm). The degree of green autofluorescence of the isolated salt glands was high since excitation intensity of as low as 8-10% was sufficient to reveal a general outline of the salt gland (Figures 4 and 5). Autofluorescence of the salt glands was also intrinsically strong with no signs of photobleaching during laser scanning.

**Figure 6:**
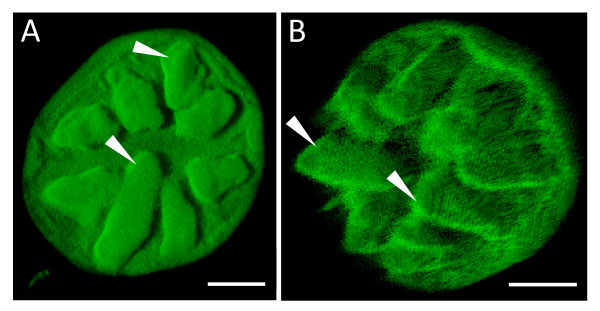
**Three-dimensional images of isolated *A*. *officinalis *salt gland**. Top view (A) and side view (B) of the same salt gland showing 8 secretory cells (arrows). Optical sections were first taken using Zeiss LSM 510. Excitation and emission wavelengths were 488 nm (12%) and >505 nm, respectively. Each three-dimensional image was constructed from the stacked images using Imaris™ (beta) 3.0. Bars = 10 μm.

**Figure 7:**
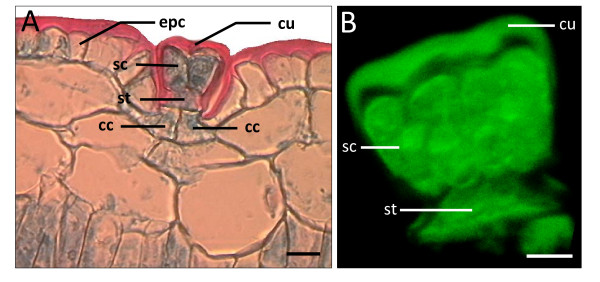
**Comparison of microtome salt gland section with isolated salt gland of *A. officinalis***. (A) Microtome section of pre-fixed paraffin-embedded leaf specimen showing cuticle envelope (cu) surrounding the salt gland and above the epidermal cells (epc). Bar = 20 μm. (B) Three-dimensional image of isolated salt gland (side view) showing 4 secretory cells (sc) above the stalk cell (st). The three-dimensional image was constructed using Imaris™ (beta) 3.0, based on stacked images captured with Zeiss LSM 510. Excitation and emission wavelengths were 488 nm (43%) and >505 nm, respectively. Bar = 10 μm. Note the presence of two collecting cells (cc) in (A) which are lacking in the isolated salt gland (B).

Autofluorescing regions were detected, mainly from the cuticular envelope, the secretory cells and the stalk cell (Figures 4, 5, 6 and 7). Optical sections of the salt gland revealed the presence of more intense green autofluorescence along the periphery of almost all the secretory cells sectioned (Figure 5). Apparent autofluorescence of spherical (Figure 5C) or irregularly shaped (Figures 4 and 5) objects was observed within the secretory cells, but not in the stalk cell. Construction of three-dimensional images of isolated salt glands was possible, with an in-depth salt gland image observed at the expense of decreased transparency (Figures 6 and 7B). It is also possible to modify the stacked images of a top view salt gland at an angle such that the z-plane information and the contours of the secretory cells were examined without having the need to capture a side view image (Figure 6B).

Autofluorescence of the salt gland cells revealed a total of 8 secretory cells, which was obvious from the three-dimensional top view image of isolated salt gland (Figure 6). A total of 2-4 secretory cells and 1 stalk cell enclosed in a cuticular envelope were noted from the side view of the isolated salt glands (Figures 4A-C, 5 and 7B). The base of the stalk cell was found to be about 25 μm wide and the other side in contact with the base of the secretory cells was only about 10 μm (Figure 7B). A subcuticular cavity (with no autofluorescence) between the cuticle envelope and the secretory cells was also observed (Figure 5 and figure 7B). Collecting cells located beneath the stalk cell of each salt gland as detected from the microtome section of paraffin-embedded leaf specimen (Figure 7A) were probably lost during isolation since such cells were not observed in the isolated salt gland (Figure 7B).

The total number of 6-8 secretory cells observed from isolated *A*. *officinalis *salt glands in this study was slightly lesser than that described previously on this genus [1,4,5,16,17]. A range of 8-12 secretory cells was reported for *Avicennia marina *salt glands [2,5], while the salt glands of *Avicennia germinans *were found to contain 8 secretory cells [8].

Confocal fluorescent microscopic studies of isolated salt glands were made possible with the intrinsic green autofluorescent properties present in the salt glands. More plant tissues were found to show background autofluorescence compared to animal tissues due to the presence of large quantities of naturally occurring fluorochromes [18]. One such fluorochrome is lignin, which is commonly synthesized and deposited in the cell walls. This compound shows an intense green fluorescence when excited with UV or blue light. Such autofluorescence usually poses a problem in fluorescent microscopy because of the overlapping spectra with other secondary fluorochromes used for research. On the other hand, autofluorescence can also be an advantage as it can serve as an internal structural marker [18-20].

**Figure 8:**
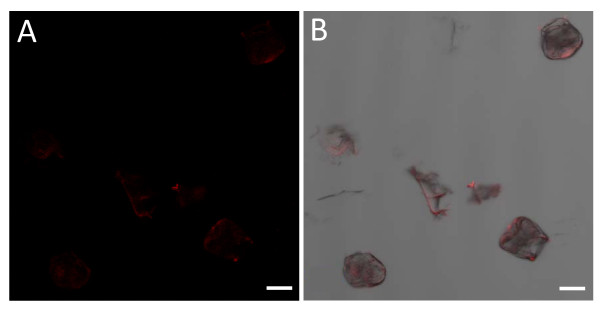
**Confocal microscopic images of isolated *A*. *officinalis *salt glands showing the degree of red autofluorescence**. (A) Image of isolated salt glands showing red autofluorescence (faintly visible). (B) Image of isolated salt glands captured simultaneously in the transmission and fluorescent modes. Excitation wavelength was 543 nm (100%) and signals in the red wavelength range were captured with Zeiss LSM 510 using high pass filter 560 nm. Bars = 20 μm.

Isolated salt glands excited with a 543 nm laser showed only faintly visible red autofluorescence when the excitation intensity was increased up to a maximum of 100% (Figure 8). Naturally occurring fluorochromes like chlorophyll were reported to produce red autofluorescence when excited by a 514 nm Ar ion laser [18,20,21], which also explains the high background fluorescence observed from the isolated epidermal peels with chlorophyll-containing mesophyll-palisade cell layers underneath. The absence of red autofluorescence in *A*. *officinalis *salt gland suggests that compounds like chlorophyll might be absent in these cells. Previous reports also revealed the absence of functional chloroplasts in all species of salt glands except for the bladder cells of *Atriplex *[1,9]. Further salt gland studies that included the use of fluorochromes with emission spectral in the red wavelength range were thus possible.

### Cytological studies of isolated salt glands with the use of various fluorochromes

**Figure 9:**
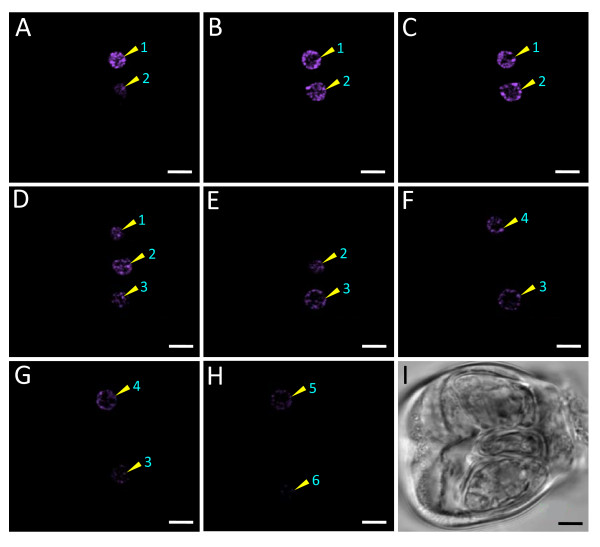
**Optical sections of a side view salt gland showing the distribution of nuclei**. (A-H) Positions of nuclei (purple pseudo-colour; 1-6) at different focal planes. (I) Corresponding bright-field image of the same salt gland under study. The salt gland was stained with Coulter^® ^DNA-Prep Stain containing 8 μg ml^-1 ^PI and images were captured using Zeiss LSM 510. Excitation and emission wavelengths were 543 nm and >560 nm, respectively. Bars = 5 μm.

**Figure 10:**
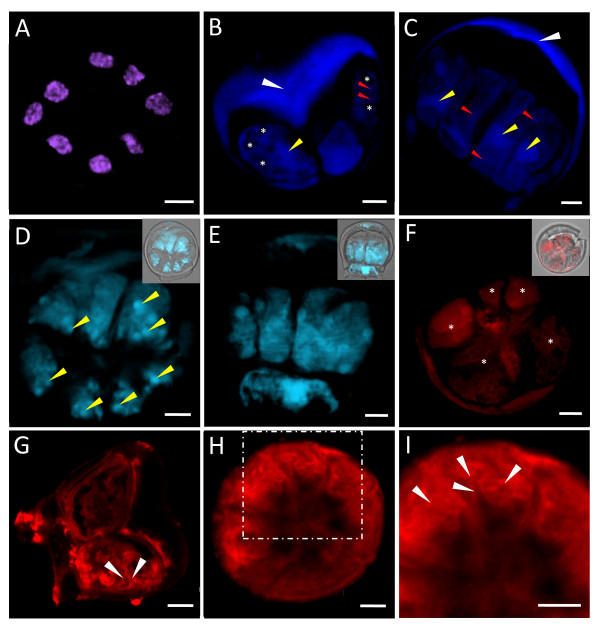
**Cytological studies of isolated salt glands of *A*. *officinalis *with the use of various fluorescent dyes**. (A) Top view of isolated salt gland treated with Coulter^® ^DNA-Prep Stain (containing 8 μg ml^-1 ^PI). (B, C) Confocal microscopic images of isolated salt glands after LysoTracker^® ^Red DND-99 (40 nM) staining. Note the relatively large (yellow arrows) and small (red arrows) internal cellular structures, which are presumably to be organelles that are acidic in nature. Prominent unstained regions (*) are observed especially in the secretory cells of (B). Non-specific staining of the cuticular envelope (white arrows) is also observed. (D, E) Confocal microscopic images of isolated salt glands stained with 6.6 nM FM^®^4-64. Intense staining of spherical-like structures (arrows) is detected within the secretory cells. Insets: corresponding image of the same salt gland captured simultaneously in the fluorescent and transmission modes. (F) Top view of isolated salt gland showing apparent staining of cells (*) after incubation in MitoTracker^® ^Red CM-H_2_XRos. Inset: corresponding bright-field image of (F) taken together with overlapping image recorded simultaneously in the transmission and fluorescent modes. Side view (G) and top view (H, I) of isolated salt glands stained with Texas Red^®^-X phalloidin showing the presence of actin-like filaments (arrows). (I) An enlarged portion of (H) with prominent actin-like filaments (arrows) along the peripheral regions. Bars = 5 μm.

Cytological studies of the isolated salt glands were conducted through the applications of fluorescent dyes viewed under confocal laser scanning microscopy (Figures 9 and 10). The choice of fluorochromes is important to avoid emission spectra that will coincide with the green autofluorescence of the salt glands. Successful staining of the salt glands with the nuclear stain propidium iodide (PI) revealed that most of the salt gland nuclei were spherical in shape (Figure 9), but occasionally elongated to irregularly shaped nuclei were also observed (Figure 10A). Non-uniform staining of the nuclei was detected in some cases, with more intensely stained dot-like structures, possibly chromatin, being evident in each nucleus of these salt glands (Figure 10A).

The secretory cells of the salt glands were mono-nucleated (Figures 9 and 10A). Each nucleus was found to be approximately 4-6 μm in diameter (Figures 9 and 10A). Relatively large nuclei of the secretory cells were also observed in other *Avicennia *species and in the salt glands of other genera [1,2,5,7,16,17]. Information on the correlation between the activity of salt gland nuclei and the activity of the salt glands, however, is lacking, even though larger nucleoli were reported in the secreting salt glands of *Limonium platyphyllum *in comparison to those without salt challenge [22].

With the availability of isolated salt glands, optical sections at different focal planes could be taken to study the distribution of organelles within the gland cells in a three-dimensional context. The location of the nuclei, for example, as revealed by optical sections of isolated salt glands stained with the nuclear stain PI, was mostly found in the middle of the secretory cells (Figure 9). Large nucleus was also reported to be located predominantly in the central region of each secretory cell in *Avicennia marina *[7]. The positions of the nuclei observed herein were also similar to the green, spherical, autofluorescent structures noted in the middle of the secretory cells of these salt glands (Figures 4 and 5). In *Nicotiana tobacum *suspension cells, the nuclei were reported to be the main organelles that showed green autofluorescence [23].

Well-distributed fluorescent images of the secretory cells were observed when the isolated salt glands were stained with LysoTracker^® ^Red DND-99 (Figure 10B, C), an acidotropic fluorescent probe that is effective in labelling typically spherical, acidic organelles by selectively accumulating itself in compartments where the pH is low [21]. Several brightly stained structures, each about 1 μm in diameter, were detected in the secretory cells of salt glands that were exposed to the dye for 60-90 min (Figure 10B, C). More intensely stained regions of the secretory cells were also observed in these glands, with their shapes and positions coinciding with the green autofluorescence structures (Figures 4 and 5) described earlier. Negatively stained regions that appeared spherical or elliptical in shape (~1.5-3.0 μm) were consistently found towards the periphery of the secretory cells (Figure 10B).

Vacuoles of plant cells are generally acidic compartments that are similar to the vacuoles of algae and fungi and do share some properties that are also present in the mammalian lysosomes. They are usually present with their internal pH being lower when compared to the cytoplasm of the cells [24,25]. Observations reported in this study suggest the presence of acidic spherical organelles in the secretory cells of salt glands. Micro-vacuoles or vesicles were also previously reported to be evenly distributed throughout the secretory cells of *Avicennia *[1,2,5,7,17].

Positive staining of the secretory cells was detected when the isolated salt glands were exposed to FM^® ^4-64 (Figure 10D, E), one of the lipophilic or amphiphilic styryl dyes that is weakly fluorescent or virtually non-fluorescent in aqueous medium but can fluoresce brightly when it is associated with or incorporated into the outer leaflet of the plasma membranes [20,21,26,27]. Developed with animal cell systems, FM^® ^4-64 was found to be nontoxic to cells and may be internalized following extended staining times [21,27-31]. The dye can thus be used as a vital stain and was reported to act as an endosomal marker in the study of endocytosis, membrane trafficking and cell division in living cells [21,26-29,32,33].

In this study, the secretory cells of FM^® ^4-64-treated salt glands were shown to fluoresce brightly, with evident cell outlines detected (Figure 10E) without the reference from the overlapping brightfield images (inset of Figure 10E). The stain was not confined to the plasma membrane, as small, dot-like structures (estimated to be 0.5-3 μm in diameter) that fluoresce brightly were detected to be consistently distributed in the secretory cells (Figure 10D, E), a sign of internalization of dyes with prolonged staining. The stalk cell of the treated salt gland was also observed to be positively stained with FM^® ^4-64, with an intensely stained spherical structure in the middle of the cell being most prominent (Figure 10E).

Taken into consideration that the secretion of salts via the salt glands had always been implied to be an active process [1,2,34], the establishment of a mitochondrial profile within the salt gland cells will provide useful information on the metabolic status of the salt glands. Attempts were made to look into the distribution of mitochondria in the gland cells using MitoTracker^® ^Red CM-H_2_XRos, a cell-permeant fluorescent dye that selectively stains mitochondria. Positive staining of the secretory cells was evident, even though MitoTracker^® ^Red CM-H_2_XRos could also non-specifically stain the cuticle layer of the isolated salt gland (Figure 10F). Fricker et al. [20] commented that this dye did not appear to stain plant mitochondria well. Nevertheless, the patches of red fluorescent signals as observed herein seemed to indicate a high mitochondrial density in the secretory cells of salt glands, which is a common observation reported by many researchers [1,2,5,7,8,17,34,35].

Establishment of the distribution of cytoskeletons in the salt glands, and their possible participation during the secretion of salts had not been reported previously in salt gland studies and will be useful in providing information towards our understanding of the secretory process. In this study, Texas Red^®^-X phalloidin was used to stain the isolated salt glands (Figure 10G-I). This dye is a fluorescent phalloidin derivative that can be used as a fluorescent probe in cytoskeletal studies due to their ability to bind to, and to a certain extent, stabilize F-actin [20,21,36]. Abundant actin-like filaments were observed in the secretory cells of isolated salt glands stained with Texas Red^®^-X phalloidin (Figure 10G-I). Such structures were found to be especially prominent on the periphery of the secretory cells towards the periclinal walls nearer to the cuticle layer (Figure 10H, I), or along the circumference of the secretory cells (Figure 10G). Some of these thread-like structures were also observed to extend towards the centre of the secretory cells, which were only visible from the highly magnified images (Figure 10G).

The use of commercially-available secondary fluorochromes to study the cellular structures of the isolated salt glands is thus possible as presented herein. Useful cytological information was obtained through this approach, with the presence of relatively large nuclei and abundant mini-vacuoles and/or vesicle-like structures and possibly abundant mitochondria in the secretory cells implying a high metabolic state of these specialized glands.

## Conclusions

In this paper, we describe a simple method to isolate large numbers of salt glands from the leaves of the mangrove species *A*. *officinalis *within a day for microscopic studies. The isolated glands exist as individual entities are useful for subsequent imaging without the interference of neighbouring leaf tissues, which tend to obscure the signal from any in-focus structural information of the salt glands. Potential applications of confocal fluorescence microscopic techniques, which have made a strong impact in plant-cell imaging [14] could also be achieved using these isolated salt glands. Optical sectioning and subsequent three-dimensional imaging of the salt glands are now technically feasible. Structural organizations of these glands can be determined from a three-dimensional perspective even without the use of secondary fluorochromes due to their intrinsic autofluorescent nature. Successful confocal microscopic imaging of isolated salt glands stained with commercially available fluorescent dyes as presented herein further provide a new approach in obtaining useful cytological information for salt gland research.

## Methods

### Isolation and purification of salt glands

Abaxial epidermal layers of excised leaves of *A*. *officinalis *were removed with razor blade and the leaves were cut into segments (2 cm by 1 cm) prior to enzyme treatment. The leaf strips floated on enzyme mixture (pH 5.7; filter-sterilized) containing 0.1% (w/v) Pectolyase Y-23 (Seishin Pharmaceutical, Japan), 1.0% (w/v) Driselase (Sigma-Aldrich, USA) and 1.0% (w/v) Cellulase Y-C (Kikkoman Corporation, Japan) were vacuum infiltrated for 10 min and incubated in the dark at 30 °C, 30 rpm for 1 h. The adaxial epidermal peels obtained were rinsed, the mesophyll-palisade layers gently scraped off using a scalpel before the peels devoid of chlorophyll-containing cells were transferred to a mortar containing 3-5 ml of 1× PBS buffer (pH 7.4). These peels were then ground to release the salt glands from the epidermal peels. The grinding process was repeated several times with fresh 1× PBS buffer (pH 7.4) to release more salt glands.

The salt gland suspensions collected in centrifuge tubes were filtered through a series of Falcon^® ^cell strainers (BD Biosciences, USA) of different pore sizes (100 μm, 70 μm followed by 40 μm) to remove the bigger cell clusters and peel debris. The isolated salt glands collected on a 20 μm cup Filcon (BD Biosciences, USA) were finally rinsed down with 2 ml of fresh 1× PBS buffer (pH 7.4) and kept on ice for subsequent observations.

### Determination of salt gland density in suspension

The density of salt glands collected in suspension was determined as the number of salt glands per cm^2 ^of leaf used. The number of salt glands was estimated with a hemocytometer. Total area of leaves used for salt gland isolation was determined with LI-3100C Area Meter (LI-COR^® ^Biosciences, USA).

### Preparation of isolated salt glands for confocal microscopic studies

Isolated salt glands in suspensions were fixed for 10 min at room temperature with freshly prepared 3.7% (w/v) paraformaldehyde dissolved in 1× PBS buffer (pH 7.4). When required, the salt glands were permeabilized with 0.1%, (v/v) Triton^® ^× 100 prepared in 1× PBS buffer (pH 7.4) for 10 min at room temperature prior to staining. The salt glands were then stained according to the manufacturer's (see Staining procedures) protocols. All dyes were removed after staining by rinsing the isolated salt glands twice with 1× PBS buffer (pH 7.4). SlowFade^® ^Light Antifade Kit (Molecular Probes, USA) was used after staining. The isolated salt glands was first pre-equilibrated in 10 drops of SlowFade^® ^Light Equilibration Buffer for 5-10 min followed by the addition of SlowFade^® ^Light antifade reagent (1-2 drops) to the sample. The salt glands were finally mounted on glass slides and sealed with nail varnish for subsequent confocal microscopic examinations.

### Staining procedures

Potential applications of various fluorescent dyes were tested on the isolated salt glands. For propidium iodide (PI) staining, isolated salt glands were permeabilized and stained for 1 h with Coulter^® ^DNA-Prep Stain (Beckman Coulter Inc, USA) diluted to a final concentration of 8 μg ml^-1 ^PI. For LysoTracker^® ^Red DND-99 staining, the glands were incubated in 40 nM LysoTracker^® ^Red DND-99 (Molecular Probes, USA) for a period of 60-90 min. FM^® ^4-64 [N-(3-triethylammoniumpropyl)-4-(6-(4-(diethylamino)phenyl) hexatrienyl)pyridinium dibromide); Molecular Probes, USA] was used according to Fischer-Parton et al. [28] and Parton et al. [29] with a final working solution of 8.25 nM. The isolated salt glands were stained for 30-90 min. For MitoTracker^® ^Red CM-H_2_XRos staining, the isolated salt glands were permeabilized before they were stained with 100 nM MitoTracker^® ^Red CM-H_2_XRos (Molecular Probes, USA) for 30 min. For Texas Red^®^-X phalloidin staining, the isolated salt glands were permeabilized prior to staining with 165 nM Texas Red^®^-X phalloidin (Molecular Probes, USA) for 20 min.

### Confocal Laser Scanning Microscopy

Observations of isolated salt glands were performed with a Zeiss LSM 510 laser scanning confocal microscope. To detect autofluorescence of samples, the unstained salt glands mounted on coverslips were excited at 488 and 543 nm. The degree of autofluorescence of the salt glands at each excited wavelength was then determined and images observed in the fluorescent and transmission modes were captured simultaneously. Where possible, optical sections of each isolated salt gland were captured and the images stacked before the three-dimensional image of the corresponding salt gland was created using Imaris™ (beta) 3.0. For isolated salt glands stained with various fluorescent dyes, the excitation wavelength for all was 543 nm and emission filter was high pass 560 nm for PI, LysoTracker^® ^Red DND-99, MitoTracker^® ^Red CM-H_2_XRos and Texas Red^®^-X phalloidin while high pass 650 nm filter was used for FM^® ^4-64.

### Light Microscopy

Tissues prepared for light microscopy were according to Ruzin (1999) with modifications. Briefly, the leaf tissues were fixed in FAA (formalin-acetic acid-alcohol), vacuum infiltrated and incubated in fixatives overnight at room temperature. They were then dehydrated in an alcohol series (50, 70, 85, 95 and 100%; at least 2 h in each solution), transferred to 100% TBA (Tert-butyl alcohol; 2 changes; at least 2 h for each incubation), incubated in a series of TBA: paraffin wax mixture (75:25, 50:50, 25:75, 0:100 two times) at 58 °C, with each incubation lasting for at least 4 h before the tissues were finally embedded in paraffin wax. Microtome sections of 10-μm thick were cut (Reichert-Jung 2030 Biocut, Germany) and sections with salt glands were selected and mounted on glass slides. The sections were stained with safranin O, covered with cover slips before they were observed under light microscope (Olympus).

## Competing interests

The authors declare that they have no competing interests.

## Authors' contributions

WKT developed the isolation method, carried out all experiments, performed sample imaging and drafted the manuscript under the close supervisions of CSL and TML. CSL participated in the overall design of the study and helped to draft the manuscript. All authors have read and approved the final manuscript.
